# Adenine Nucleotide Translocator Transports Haem Precursors into Mitochondria

**DOI:** 10.1371/journal.pone.0003070

**Published:** 2008-08-27

**Authors:** Motoki Azuma, Yasuaki Kabe, Chikanori Kuramori, Masao Kondo, Yuki Yamaguchi, Hiroshi Handa

**Affiliations:** 1 Department of Biological Information, Graduate School of Bioscience and Biotechnology, Tokyo Institute of Technology, Midori-ku, Yokohama, Japan; 2 Department of Integrative Medical Biology, School of Medicine, Keio University, Shinjuku-ku, Tokyo, Japan; 3 Department of Early Childhood Care and Education, Toyoko Gakuen Women's College, , Setagaya-ku, Tokyo, Japan; 4 Solutions Research Organization, Integrated Research Institute, Tokyo Institute of Technology, Midori-ku, Yokohama, Japan; Baylor College of Medicine, United States of America

## Abstract

Haem is a prosthetic group for haem proteins, which play an essential role in oxygen transport, respiration, signal transduction, and detoxification. In haem biosynthesis, the haem precursor protoporphyrin IX (PP IX) must be accumulated into the mitochondrial matrix across the inner membrane, but its mechanism is largely unclear. Here we show that adenine nucleotide translocator (ANT), the inner membrane transporter, contributes to haem biosynthesis by facilitating mitochondrial accumulation of its precursors. We identified that haem and PP IX specifically bind to ANT. Mitochondrial uptake of PP IX was inhibited by ADP, a known substrate of ANT. Conversely, ADP uptake into mitochondria was competitively inhibited by haem and its precursors, suggesting that haem-related porphyrins are accumulated into mitochondria via ANT. Furthermore, disruption of the ANT genes in yeast resulted in a reduction of haem biosynthesis by blocking the translocation of haem precursors into the matrix. Our results represent a new model that ANT plays a crucial role in haem biosynthesis by facilitating accumulation of its precursors into the mitochondrial matrix.

## Introduction

Haem, an endogenous porphyrin derivative, is a prosthetic group for haem proteins, which play an essential role in oxygen transport, respiration, signal transduction, and detoxification. Haem biosynthesis is catalyzed by eight enzymes. The porphyrin ring structure is generated in the cytosol, and then the haem precursor protoporphyrinogen IX is transported into the intermembrane space of mitochondria. PP IX is generated from protoporphyrinogen IX by protoporphyrinogen oxidase (PPO) on the outer surface of the mitochondrial inner membrane. Finally, haem is generated by ferrochelatase (FECH)-catalyzed iron insertion into PP IX on the inner surface of the mitochondrial inner membrane [1].

Mitochondria are enclosed by two layers of membranes, the outer membrane and the inner membrane. In haem biosynthesis, haem precursors must be transported into the matrix across the two membranes. ABCB6 and PBR are candidate porphyrin transporters across the outer membrane [2], [3]. Particularly, ABCB6 is thought to play a crucial role in haem biosynthesis by facilitating the accumulation of porphyrins into mitochondria [3]. On the other hand, it is controversial how PP IX reaches the mitochondrial matrix through the inner membrane. It has been suggested that PPO binds to FECH across the inner membrane, and that this association permits the direct transfer of PP IX from PPO to FECH [4]. However, Proulx *et al.* reported that the PPO/FECH complex formation is not essential for haem biosynthesis, because exogenous PP IX could be converted into haem independently of the PPO/FECH complex [5]. It is therefore likely that there is another inner membrane transport system for PP IX.

We have studied on the mitochondrial accumulation of phosphorescent porphyrin derivatives. Using affinity latex beads, the mitochondrial transporter 2-oxoglutarate carrier (OGC) was identified as a protein binding to phosphorescent porphyrins and was shown to be responsible for the accumulation of phosphorescent porphyrins in mitochondria [6]. However, it remains unclear whether OGC has any effect on the transport of natural porphyrins and on haem biosynthesis. Here, we identified ANT as a novel mitochondrial protein binding to haem and its precursors. ANT is an inner membrane transporter that facilitates the exchange of ATP and ADP [7]. We show evidence that ANT contributes to haem biosynthesis by transporting its precursors into mitochondria.

## Results

### Haem and PP IX directly bind to ANT

**Figure 1:**
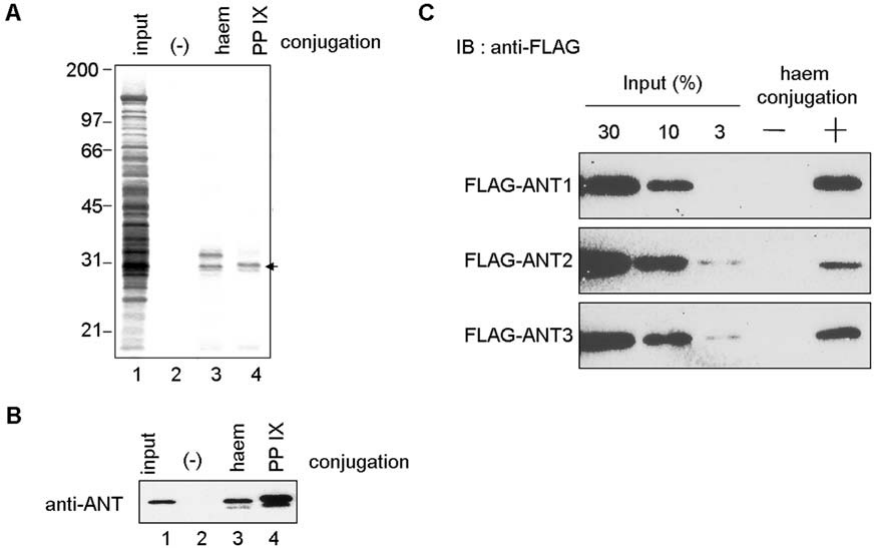
Identification of mitochodrial haem-binding proteins. Purification of haem or PP IX-binding proteins. Haem- or PP IX-conjugated or unconjugated SG beads were incubated with mitochondrial extracts of rat liver. Bound proteins were eluted with Laemmli dye and subjected to SDS-PAGE, followed by silver staining (A) or Western blotting with anti-ANT antibody (B). C, FLAG-tagged ANT1, 2, or 3 was incubated with haem-conjugated (+) or unconjugated (−) SG beads. The eluates were separated by SDS-PAGE, followed by western blotting.

First, we attempted to identify haem- or PP IX-binding proteins in mitochondria using affinity beads termed as SG beads. Carboxyl groups of haem or PP IX were succinated and conjugated to amino-modified SG beads. Using haem- or PP IX-conjugated beads, haem- or PP IX-binding proteins were purified from rat mitochondrial extract. Two proteins with apparent molecular weights of 33 and 30 kDa bound to haem-conjugated beads, while only the 30 kDa protein was found associated with PP IX-conjugated beads (Fig. 1*A*). Although the 33 kDa protein was not identified successfully, the 30 kDa protein was identified as ANT2 by Q-TOF MS analysis. The identity was confirmed by Western blotting (Fig. 1*B*). We examined the haem-binding activity of three ANT isoforms (ANT1, 2 and 3) using recombinant proteins and found that haem bound to all the ANT isoforms similarly (Fig. 1*C*). The amount of recombinant protein that bound to the haem-conjugated beads was calculated to be 10% of the input amount for ANT1, 2, and 3. We also showed that apo-myoglobin bound to the haem-conjugated beads, but that glutathione-*S*-transferase did not (Fig. S1).

### Modeling of the binding between haem and ANT

**Figure 2:**
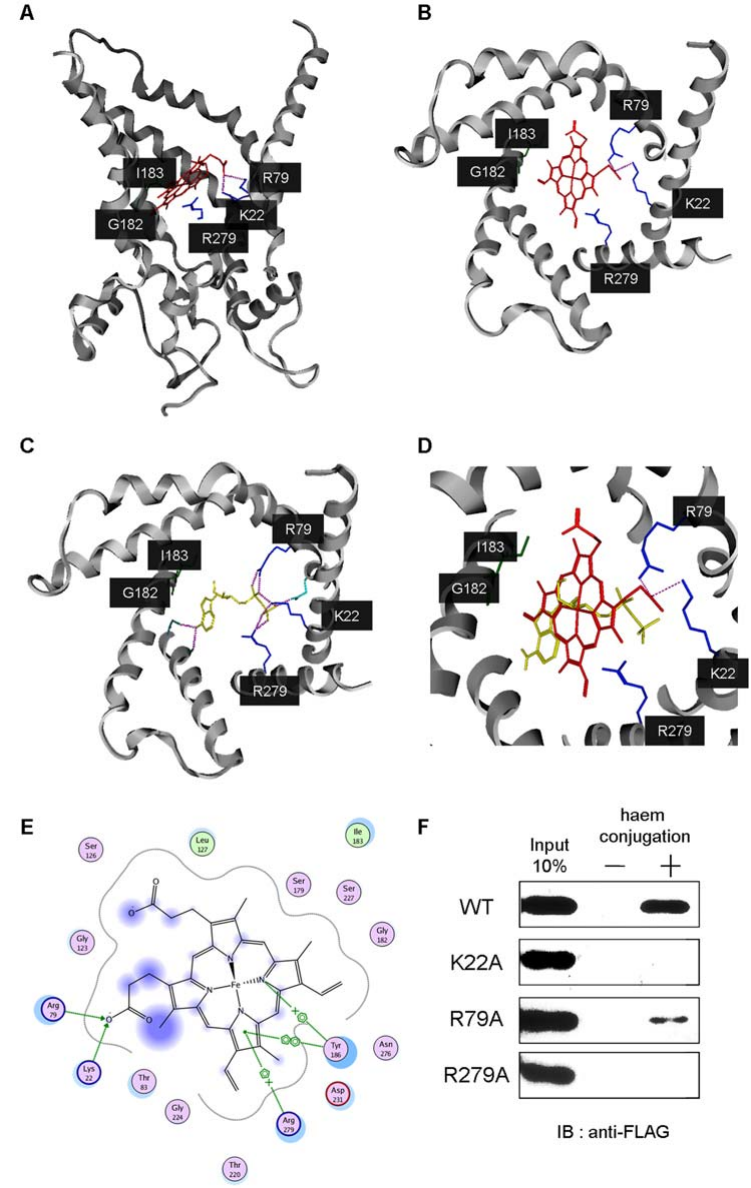
Docking model of ANT with haem. (A) Lateral view of BtAAC1 structure docking with haem (red). Blue lines highlight the contact residues of BtAAC with haem. View of the transmembrane sector from the mitochondrial intermembrane space of BtAAC1 bound to haem (red) (B) or ADP (yellow) (C). (D) A superposition of ADP and haem on BtAAC. (E) The closed conformation of BtAAC1 with haem. (F) FLAG-tagged ANT1 wild type (WT) or series of the mutant of K22A, R79A and R279A, was incubated with haem-conjugated (+) or unconjugated (−) SG beads. The eluates were separated by SDS-PAGE, followed by western blotting.

ANT is a mitochondrial carrier that transports ADP across the inner mitochondrial membrane. Recently, an atomic model of the complex between *Bos taurus* ANT1 (BtAAC1) and the ANT inhibitor carboxyatractyloside (CATR) has been proposed [8]. To elucidate the binding properties of haem for ANT, we used computational analysis based on the BtAAC1 structure. As shown in Fig. 2*A and B*, haem docked in the center pore domain of BtAAC1. Furthermore, we also showed that the ADP binding site was formed by K22, R79 and R279 residues which associate with the phosphate moieties of ADP. The residues between G182 and I183 associated with the adenine ring structure (Fig. 2*C*), as seen in the previous *in silico* analysis [9]. Interestingly, haem also associated with the K22 and R79 residues of BtAAC1 via its carboxy residue and with I183 and R279 residues via its pyrrol ring structures (Fig. 2*D and E*). To confirm the binding model, we elucidated the haem-binding activity of ANT1 mutated in the residues of K22, R79 or R279. As shown in Fig. 2*F*, while ANT1 R79A mutant remained the haem-binding activity, K22A and R279A could not bind to haem. These results strongly support our docking model between haem and ANT1.

### Haem and its precursors accumulate into mitochondria via ANT

**Figure 3:**
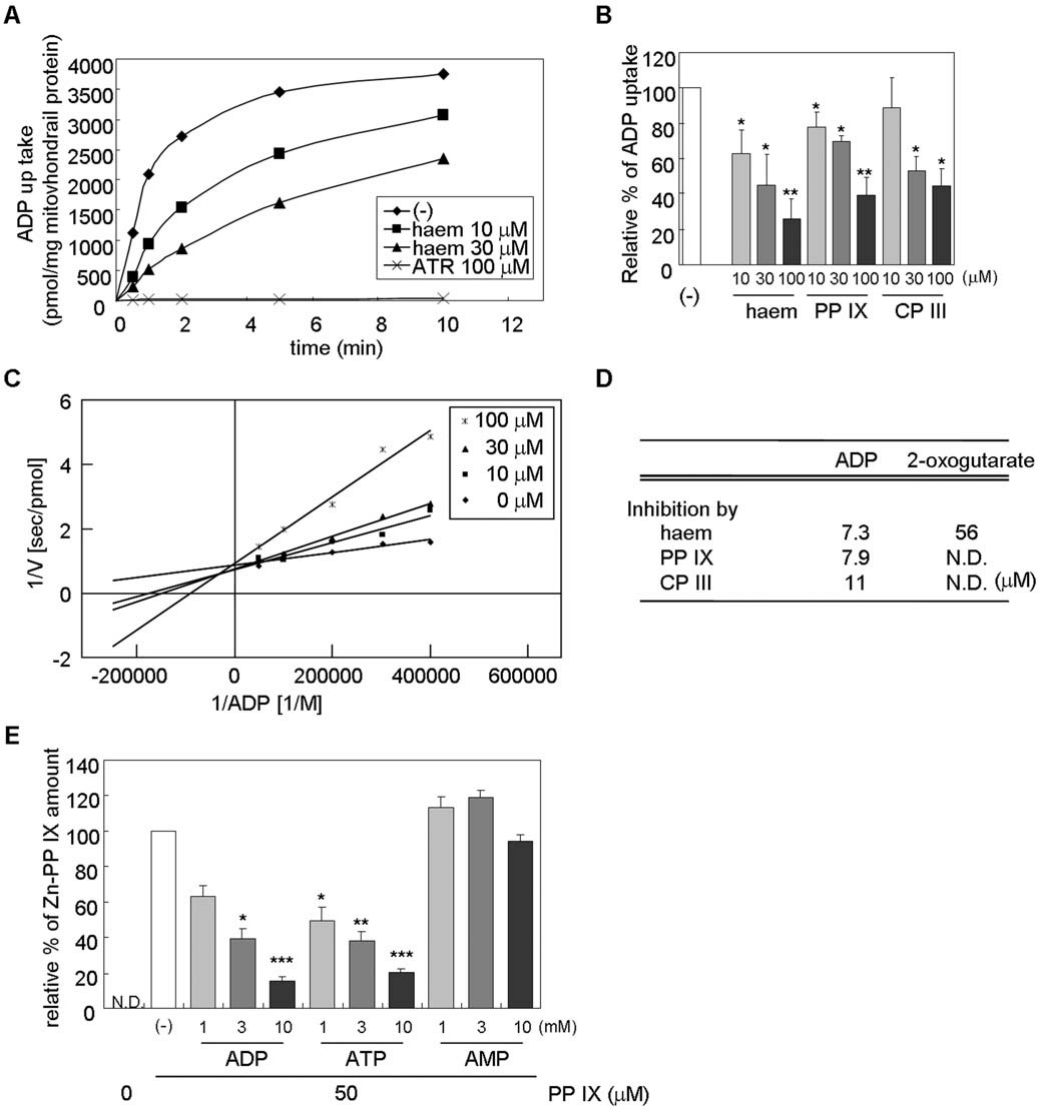
Haem and its precursors accumulate into mitochondria via ANT. (A) [^3^H]-labeled ADP was incubated with rat liver mitochondria in the presence or absence of haem or atractyloside for 0.5, 1, 2, 5, 10 min, and then the reaction was stopped by addition of atractyloside. (B) [^3^H]-labeled ADP was incubated with rat liver mitochondria in the presence of the indicated concentrations of haem, PP IX, or CP III on ice for 30 sec, and then the reaction was stopped by addition of atractyloside. (C) Lineweaver-Burk plot of ADP uptake into mitochondria in the presence of the indicated concentrations of haem for 30 sec. (D) Inhibition constant (K_i_) for the inhibition of the mitochondria uptake of ADP or 2-oxoglutarate by haem, PP IX, or CP III. N.D., cannot be detected. (E) PP IX (50 µM) and Zn-acetate (50 µM) was incubated with mitochondria in the presence or absence of ADP, ATP or AMP on ice for 30 sec The generated Zn-PP IX was extract and detected by HPLC equipped with a fluorometric detector. Data represent mean±s.e.m. from four to six independent experiments (*, *P*<0.05; **, *P*<0.01; ***, *P*<0.005).

Since the above analysis predicted that haem binds to ANT though a common ADP binding site, we hypothesized that ANT would contribute to the mitochondrial transport of haem as well as that of ADP. To elucidate this hypothesis, we analyzed the effect of porphyrins on the mitochondrial uptake of ADP using isolated rat liver mitochondria. [^3^H] labeled-ADP uptake into mitochondria reached saturation after 5 min, whereas pretreatment of atractyloside (ATR) blocked its uptake completely. Notably, haem inhibited an initial rate of ADP uptake (Fig. 3*A*). Furthermore, PP IX and CP III also strongly inhibited ADP uptake in a dose-dependent manner (Fig. 3*B*). In addition, ADP uptake was not changed by treatment with FCCP or NaN_3_, or by omitting succinate (Fig. S2 *A*). We also confirmed the disruption of membrane potential or loss of oxygen consumption by treatment with FCCP or NaN_3_, respectively (Fig. S3 and Text. S1). Lineweaver-Burk plot analysis revealed that the inhibition of ADP uptake by haem is competitive with an inhibition constant (K_i_) of 7.3 µM (Fig. 3*C and 3D*). PP IX or CP III also inhibited ADP uptake in a competitive manner with similar Ki values (Fig. 3*D*). We previously reported that haem binds to the transporter OGC and inhibits the mitochondrial uptake of its substrate, 2-oxoglutarate [6]. We therefore compared Ki for the inhibition of ADP or 2-oxoglutarate uptake by haem. As a result, the inhibitory effect of haem was found to be more potent on ADP uptake than on 2-oxoglutarate uptake, suggesting that haem and haem precursors are preferentially accumulated in the matrix by ANT.

Next, to examine the mitochondrial uptake of haem precursor PP IX, we analyzed the Zn-PP IX formation in the isolated mitochondria. PP IX and Zn-acetate were incubated with rat liver mitochondria, and then Zn-PP IX was detected by HPLC analysis. Zn-PP IX generation was significantly decreased by the addition of ADP or ATP (Fig. 3*E*), whereas it was not changed by treatment with AMP, FCCP or NaN_3_, or by omitting succinate (Fig. 3E, Fig. S2 *B*). These results suggested that PP IX is transported into the mitochondrial matrix along the same route as ADP.

### Haem biosynthesis is reduced in ANT-deficient yeast

**Figure 4:**
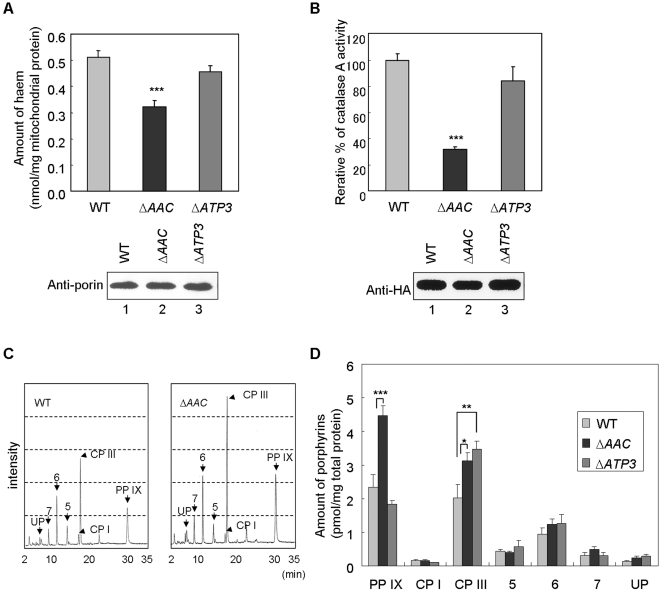
Analysis of haem biosynthesis in the ANT-deficient yeast strain. (A) The amount of mitochondrial haem in the wild-type (WT), Δ*AAC* or Δ*ATP3* yeast strain was measured with a fluorometric detector as described in *Materials and Methods*. Lower panel shows Western blot analysis of mitochondrial extracts using anti-porin antibody. (B) HA-tagged catalase A activity of the WT, Δ*AAC* and Δ*ATP3* yeast strain. HA-catalase A was immunoprecipitated, and its catalase activity was determined (upper panel). Western blotting was performed using anti-HA antibody (lower panel). (C) Haem precursors (UP, uroporphyrin; 7, heptaporphyrin; 6, hexaporphyrin; 5, pentaporphyrin; CP I, coproporphyrin I; CP III, coproporphyrin III; PP IX, protoporphyrin IX) obtained from the WT, Δ*AAC* or Δ*ATP3* yeast strain were analyzed by C_18_ reverse phase HPLC. (D) Quantification of (C). Data represent mean±s.e.m. from five independent experiments (*, *P*<0.05; **, *P*<0.01; ***, *P*<0.005).

We examined the effect of ANT on haem biosynthesis *in vivo* using an ANT-deficient yeast strain (Δ*AAC*), in which all the ANT homologous genes, *AAC1*, *AAC2*, and *AAC3*, were disrupted [10]. We found using affinity beads that Aac1p, Aac2p, and Aac3p all bind to haem (data not shown). In the Δ*AAC* strain, the amount of mitochondrial haem was found to be significantly decreased compared to the wild-type strain (Fig. 4*A*). Furthermore, we investigated whether exogenous expressed catalase A activity, which is known as haem protein, is affected in the Δ*AAC* strain. HA-tagged catalase A was immunoprecipitated, and its activity was determined. As shown in Fig. 4*B*, HA-catalase A activity was significantly reduced in the Δ*AAC* strain. These results indicated that ANT contributes to haem biosynthesis.

If the haem reduction is due to defects of the mitochondrial translocation of haem precursors, their accumulation should be observed in the Δ*AAC* strain. To examine this hypothesis, we extracted haem precursors from the wild-type and Δ*AAC* strains, followed by HPLC analysis. As shown in Fig. 4*C and D*, PP IX and CP III were dramatically increased in the Δ*AAC* strain while uroporphyrin, coproporphyrin I, and other haem precursors were not. In addition, peaks appearing at 260 nm were the same in the different yeast strains (Fig. S4). Furthermore, we also examined haem biosynthesis in an ATP synthase component ATP3-deficient yeast strain (Δ*ATP3*) [11]. As shown in Figure 4*A and B*, the amount of mitochondrial haem and catalse A activity was the same in the Δ*ATP3* yeast strain as in the wild type. Also, whereas CP III accumulation was observed in the Δ*ATP3* yeast strain, the amount of PP IX was the same as in the wild type. Although the reason for CP III accumulation is unclear, we conclude that the mitochondrial transport of the haem precursor is not impaired in the Δ*ATP3* yeast strain, since PP IX must be transported into mitochondria for haem biosynthesis. These results strongly support our hypothesis that ANT contributes to haem biosynthesis by accumulating haem precursors independently of energy production or membrane potential in mitochondria. In addition, we also analyzed haem biosynthesis using an OGC-deficient yeast strain (Δ*ODC*), in which OGC homologous genes, *ODC1* and *ODC2*, were disrupted [12]. Neither haem reduction nor accumulation of haem precursors was observed in the Δ*ODC* strain (Fig. S5). These results suggested that ANT, but not OGC, contributes to haem biosynthesis and the translocation of haem precursors into the matrix.

## Discussion

In the present study, we identified ANT as a haem or PPIX binding protein by affinity purification (Fig. 1). *In vitro* biochemical analyses revealed that ADP uptake was also inhibited by haem-related porphyrins in a competitive manner (Fig. 3). Furthermore, PP IX uptake into the mitochondrial matrix was inhibited specifically by the addition of ADP (Fig. 3*E*). These results indicate that haem and ADP are transported into mitochondria through a common pathway comprising ANT. In support of this, haem associates with the center pore site of BtAAC1, as shown by by *in silico* docking analysis (Fig. 2). Interestingly, haem makes contacts with BtAAC1 through the same residues (K22, R79, I183, R279) of ANT as those used for ADP docking. A previous study showed that mutation of residues K22, R79 or R279 abolished the transport activity of the yeast ANT [13], [14]. X-ray crystallography analysis of BtAAC1 also suggested that these residues are important for ADP binding and would contribute to its transport into the inner matrix [8]. We also showed that mutation of ANT1 K22A and R279A resulted in the disruption of its binding activity with haem (Fig. 2*F*). Taken together, these results show that haem and its precursors are incorporated into mitochondria in the same way as ADP via ANT. On the other hand, atractyloside did not affect the Zn-PP IX formation, whereas ADP or ATP inhibited strongly (Fig. S2 *B*). Although the detailed mechanism is not elucidated yet, there are several differences of the ANT-binding site between haem and atractyloside. It has been known that atractyloside interacts with R79, N87, K91, L127, V130, I183, R187, D231 and R234 of ANT1 from an atomic model of the complex [8], while haem interacts with K22, R79 G182, I183 and R279 of ANT1 in our docking analysis (Fig. 2). ANT1 R79A mutant, which overlaps the binding site with haem or atractyloside, remained the haem-binding activity, but K22A and R279, the haem-specific binding residues, disrupted its binding completely. Haem might be transported into the matrix in the presence of atractyloside through recognizing the different ANT1 residues from the atractyloside-binding site.

We previously found that OGC binds to phosphorescent porphyrin derivatives and accumulates them into mitochondria. *In vitro* analysis showed that the uptake of the ANT substrate ADP was more severely impaired by haem than the uptake of the OGC substrate 2-oxoglutarate (Fig. 3*D*). On the contrary, 2-oxoglutarate uptake was inhibited more strongly than ADP uptake by phosphorescent porphyrin derivatives such as palladium *meso*-tetra(4-carboxyphenyl)porphyrin (PdTCPP) and palladium *meso*-tetra(4-aminophenyl)porphyrin (PdTAPP) in a competitive manner, with Ki constants of 15 µM and 1.9 µM, respectively (data not shown). Moreover, neither haem reduction nor haem precursor accumulation was detected in OGC-deficient yeast. These findings suggest that ANT is primarily involved in the transport of haem precursors and haem biosynthesis, while OGC is involved in the mitochondrial accumulation of synthetic phosphorescent porphyrins. ANT may recognize the haem-related porphyrin structure, while OGC may bind to the tetraphenyl-porphyrin structure.

We showed that the disruption of ANT homologues in yeast resulted in a 40% reduction in haem content and a two-fold increase in the level of PP IX (Fig. 4). On the other hand, ATP synthase component ATP3-deficient yeast had the same levels of haem and PP IX as the wild-type strain. This indicates that ANT contributes to haem biosynthesis by accumulating the haem precursor in mitochondria independently of energy production or metabolic events. On the other hand, CP III accumulation was observed in ATP3-deficient yeast, but amount of PP IX and haem was not changed. However, the detailed mechanism is not elucidated yet, the CP III accumulation was observed commonly in ANT- or ATP3-deficient yeast, both which were abolished the energy production of mitochondria. CP III amount might be affected by energetic status of mitochondria, but haem or PP IX amount would not. Furthermore, we also showed that haem and ADP uptake in mitochondria was not affected by NaN3 or the uncoupling reagent FCCP, or by the omission of the electron transport substrate succinate (Fig S2). This suggested that the inner mitochondrial membrane potential is not necessary for haem or ADP transport. Previously, it has been suggested that PP IX accumulation in the mitochondrial matrix is mediated by the PPO/FECH transmembrane complex. However, our results strongly suggest that ANT contributes to haem biosynthesis by serving as an alternative PP IX transport pathway.

## Materials and Methods

### Materials

Haemin, PP IX, and CP III were purchased from Porphyrin Products. Anti-ANT antibody was kindly provided by Dr. Catherine Brenner-Jan [15]. Anti-FLAG antibody (M2), anti-FLAG resin, FLAG peptide, anti-HA antibody (HA-7), and anti-HA resin were purchased from Sigma.

### Plasmids construction and yeast strains

cDNAs of human *ANT1*, *ANT2*, and *ANT3* were obtained from a HL60 cDNA library. ANT1 mutagenic primers were showed in Table S1. For expression of FLAG epitope tagged ANT, the cDNAs were inserted into the mammalian expression vector pCMV-tag2. cDNA of yeast catalase A was obtained from W303-1B genomic DNA. For expression of catalase A with a carboxyl-terminal HA epitope, the cDNA was inserted into the yeast expression vector pRS424 carrying the TFA1 promoter.

Δ*AAC* yeast strain (*MAT*α *ade2-1 trp1-1 can1-100 aac3::URA3*, *aac1::LEU2*, *aac2::HIS3*) and its parent strain (W303-1B, *MAT*α *ade2-1 his3-11*, *15 lue2-3*, *112 trp1-1 ura3-1 can1-100*) were kindly provided by Dr. Yasuo Shinohara [10]. Δ*ATP3* yeast strain (W303ΔATP3, ρ^+^ *MAT*α *ade2-1 his3-11*, *15 lue2-3*, *112 trp1-1 ura3-1 can1-100 atp3::HIS3*) was kindly provided by Dr. Alexander Tzagoloff [11]. Δ*ODC* yeast strain (*MAT*a *ade2-101 lue2-Δ1 ura3-52 lys2-801 odc1::TRP1*, *odc2::HIS3*) and its parent strain (YPH499, *MAT*a *ade2-101 his3-Δ200 lue2-Δ1 trp1-Δ63 ura3-52 lys2-801*) were kindly provided by Dr. Ferdinando Palmieri [12]. Yeast cells were grown in YPD medium.

### Purification of haem binding proteins

Amino-modified SGNEGDEN beads were prepared as described [16]. To generate haem- or PP IX-conjugated beads, 0.3 mM haem or PP IX was succinated with an equal amount of N-hydroxysuccinimide in dimethylformamide and then reacted with SGNEGDEN beads at room temperature overnight as described [6].

### Preparation of recombinant proteins

293FT cells were maintained in Dulbecco's modified Eagle's medium (Invitrogen) supplemented with 10% fetal bovine serum. The expression vectors for ANT1, 2, 3 and ANT1 mutants were transfected using Lipofectamine 2000 (Invitrogen) according to the manufacturer's instructions. After 24 h, cells were harvested and lysed with NP-40 lysis buffer (20 mM Tris-HCl (pH 8.0), 150 mM NaCl, 1 mM EDTA, 1% Nonidet P-40). FLAG-tagged ANT proteins were immunoprecipitated with anti-FLAG resin and eluted with 0.1 mg/ml FLAG peptide.

### Measurement of ANT activity

Measurement of ADP uptake in mitochondria was performed as described previously [17]. Rat liver mitochondria (1 mg/ml) were pretreated with or without haem, PP IX, or CP III for 5 min in buffer (10 mM Tris-HCl (pH 7.4), 250 mM sucrose, 5 mM succinate, and 10 µM EGTA) and then incubated with ^3^H-labeled ADP (2.5–20 µM) on ice for 0.5, 1, 2, 5, 10 min. The reaction was terminated by the addition of 100 µM atractyloside (final concentration) and centrifuged at 10,000× g for 2 min at 4°C. Mitochondria were lysed with 50 µl of 1% SDS, and [^3^H] ADP uptake was measured using a liquid scintillation counter [17]. The mitochondrial uptake of 2-oxoglutarate was measured as described in Kabe *et al.* (2006) [6].

For analysis of Zn-PP IX formation in the isolated mitochondria, PP IX (50 µM) was incubated with 0.5 ml rat liver mitochondria (1 mg/ml) in the presence or absence of ADP on ice for 30 sec in buffer (10 mM Tris-HCl (pH 7.4), 250 mM sucrose, 5 mM succinate, 10 µM EGTA and 50 µM Zn-acetate) and then centrifuged at 10,000 ×g for 2 min at 4°C. The generated Zn-PP IX was extract with 1 ml ethyl acetate/acetic acid (3/1, v/v). After centrifugation at 5000 rpm for 10 min, the 10 µl supernatants were subjected to chromatography on a C_18_ reverse-phase HPLC column equipped with a fluorometric detector [21].

### Measurements of haem and its precursors in yeast

Yeast mitochondria were isolated as described in Daum *et al.* (1982) [18]. The mitochondrial haem was converted to PP IX in 2 M oxalic acid at 100°C for 30 min, and the amount of PP IX was determined by its fluorescence intensity at 600 nm [19], [20].

To detect haem precursors in yeast, yeast were grown in 100 ml of YPD medium overnight. Yeast were recovered by centrifugation at 160 ×g for 5 min, and spheroplasts were prepared as described previously [18], and then homogenated with 5 ml ethyl acetate/acetic acid (3/1, v/v). After centrifugation at 5000 rpm for 10 min, the supernatant was concentrated to 1 ml, and 10 µl samples were subjected to chromatography on a C_18_ reverse-phase HPLC column equipped with a fluorometric detector [21].

### Measurement of catalase activity

Yeast cells transformed with an expression vector of HA-tagged catalase A were harvested and lysed with glass beads in lysis buffer (50 mM potassium phosphate, 1 mM EDTA). After centrifugation at 15,000 rpm for 15 min at 4°C, HA-tagged catalase A was purified from the supernatant with anti-HA resin. Catalase activity was measured using Catalase Assay Kit (Cayman Chemical) according to the manufacturer's instructions.

### Docking analysis between haem and ANT1

*In silico* work was performed on a Intel® Pentium® D (2.80 GHz) Windows Vista™ Business computer with the Molecular Operating Environment™ (MOE, Version 2007.0902) software developed by the Chemical Computing Group Inc., Montreal, Canada. Using MOE, we attempted to model haem-bovine ANT (PDB code: 1OKC [8]) protein docking. At first, hydrogen atoms were added and forcefield (MMFF94x [22]–[24] for ADP and ANT, CHARMM22 [25] for haem) atomic charges were assigned. Then, MOE-ASEDock 2005 [26] was used for the docking of ADP or haem to ANT.

## Supporting Information

Figure S1 Purification of apo-myoglobin or glutathione-S-transferase using haem-conjugated beads. 1 µg of Apo-myoglobin (A) or glutathione-S-transferase (GST) (B) was incubate with haem-conjugated (+) or unconjugated (−) SG beads. The eluates were separated by SDS-PAGE, followed by silver staining (A) or western blotting with anti-GST antibody (B). Equine apo-myoglobin was prepared as described previously [27].
(1.71 MB TIF)

Figure S2 Analysis of haem or ADP uptake into mitochondria. (A) [3H]-labeled ADP was incubated with rat liver mitochondria in the presence of FCCP (1 µg/ml), NaN3 (5 mM) or haem (100 µM) or in the absence of succinate on ice for 30 sec. (B) PP IX (50 µM) and Zn-acetate (50 µM) was incubated with rat liver mitochondria in the presence of FCCP (1 µg/ml), NaN3 (5 mM), atractyloside (100 µM) or ADP (10 mM) or in the absence of succinate on ice for 30 sec. The generated Zn-PP IX was extract and detected by HPLC equipped with a fluorometric detector. Data represent mean±s.e.m. from four to six independent experiments (**, P<0.01; *** P<0.005).
(3.79 MB TIF)

Figure S3 Analysis of mitochondria activity. (A) The membrane potential of mitochondria was determined by incorporation of Rhodamine 123 (Rho 123). The percentage of mitochondria above a fluorescence threshold is shown at the top right corner of each panel. (B) After incubation of mitochondria (mito) with 5 mM NaN3 for 5 min, ADP was added, and oxygen concentration in solution was measured realtime.
(5.64 MB TIF)

Figure S4 Absorbance of extracts from the WT or ΔAAC yeast strain. Extracts from the WT or ΔAAC yeast strain used in Fig. 4C were analyzed by C18 reverse phase HPLC and the absorbance of the eluted fractions was measured at 260 nm.
(1.47 MB TIF)

Figure S5 Analysis of haem biosynthesis in the OGC-deficient yeast strain. (A) The amount of mitochondrial haem in the wild-type (WT) or ΔODC yeast strain was measured with a fluorometric detector as described in Materials and Methods. Lower panel shows Western blot analysis of mitochondrial extracts using anti-porin antibody. (B) The concentrations of haem precursors (UP, uroporphyrin; 7, heptaporphyrin; 6, hexaporphyrin; 5, pentaporphyrin; CP I, coproporphyrin I; CP III, coproporphyrin III; PP IX, protoporphyrin IX) in the WT or ΔODC yeast strain. Data represent mean±s.e.m. from five independent experiments.
(2.47 MB TIF)

Table S1 (1.56 MB TIF)

Text S1 Supporting Method S1.
(0.03 MB DOC)
