# Supplementary figures and images for: Adenine Nucleotide Translocator Transports Haem Precursors into Mitochondria
Source: PLoS One. 2008 Aug 27;3(8):e3070. doi: 10.1371/journal.pone.0003070 (PMC2516936; doi:10.1371/journal.pone.0003070)

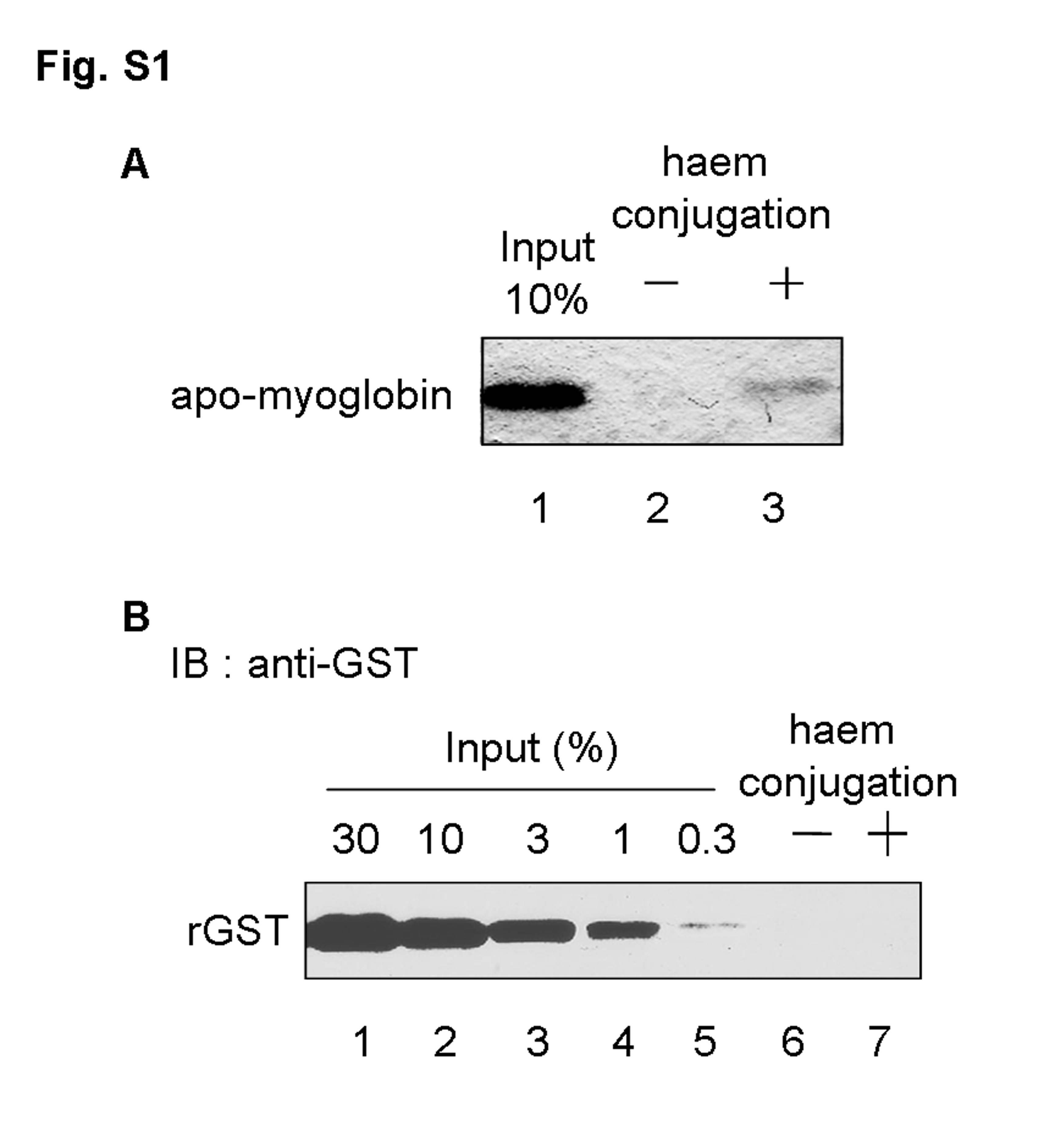

Supplement: Figure S1 — Purification of apo-myoglobin or glutathione-S-transferase using haem-conjugated beads. 1 µg of Apo-myoglobin (A) or glutathione-S-transferase (GST) (B) was incubate with haem-conjugated (+) or unconjugated (−) SG beads. The eluates were separated by SDS-PAGE, followed by silver staining (A) or western blotting with anti-GST antibody (B). Equine apo-myoglobin was prepared as described previously [27]. (1.71 MB TIF) [file pone.0003070.s001.tif]

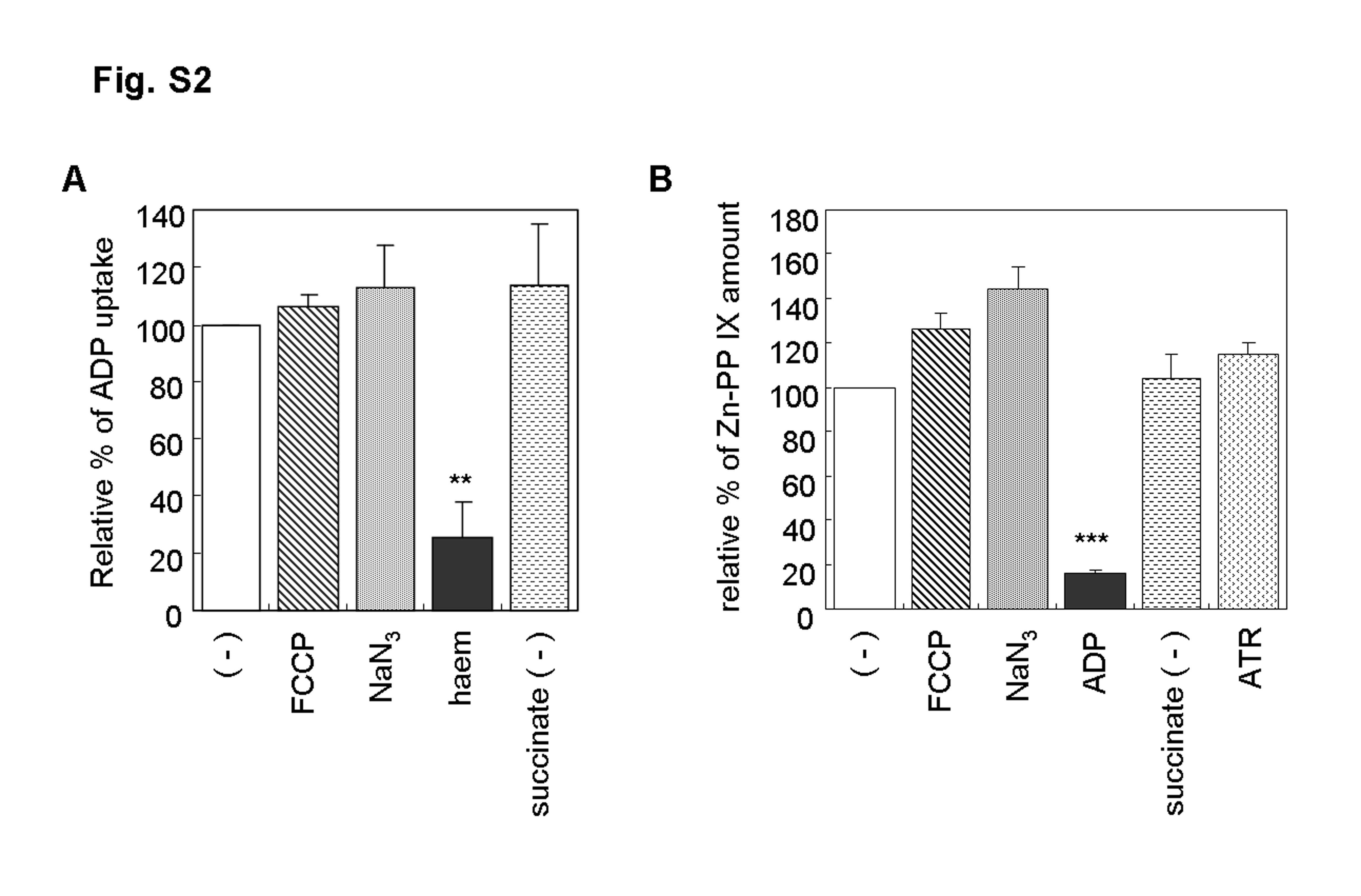

Supplement: Figure S2 — Analysis of haem or ADP uptake into mitochondria. (A) [3H]-labeled ADP was incubated with rat liver mitochondria in the presence of FCCP (1 µg/ml), NaN3 (5 mM) or haem (100 µM) or in the absence of succinate on ice for 30 sec. (B) PP IX (50 µM) and Zn-acetate (50 µM) was incubated with rat liver mitochondria in the presence of FCCP (1 µg/ml), NaN3 (5 mM), atractyloside (100 µM) or ADP (10 mM) or in the absence of succinate on ice for 30 sec. The generated Zn-PP IX was extract and detected by HPLC equipped with a fluorometric detector. Data represent mean±s.e.m. from four to six independent experiments (**, P<0.01; *** P<0.005). (3.79 MB TIF) [file pone.0003070.s002.tif]

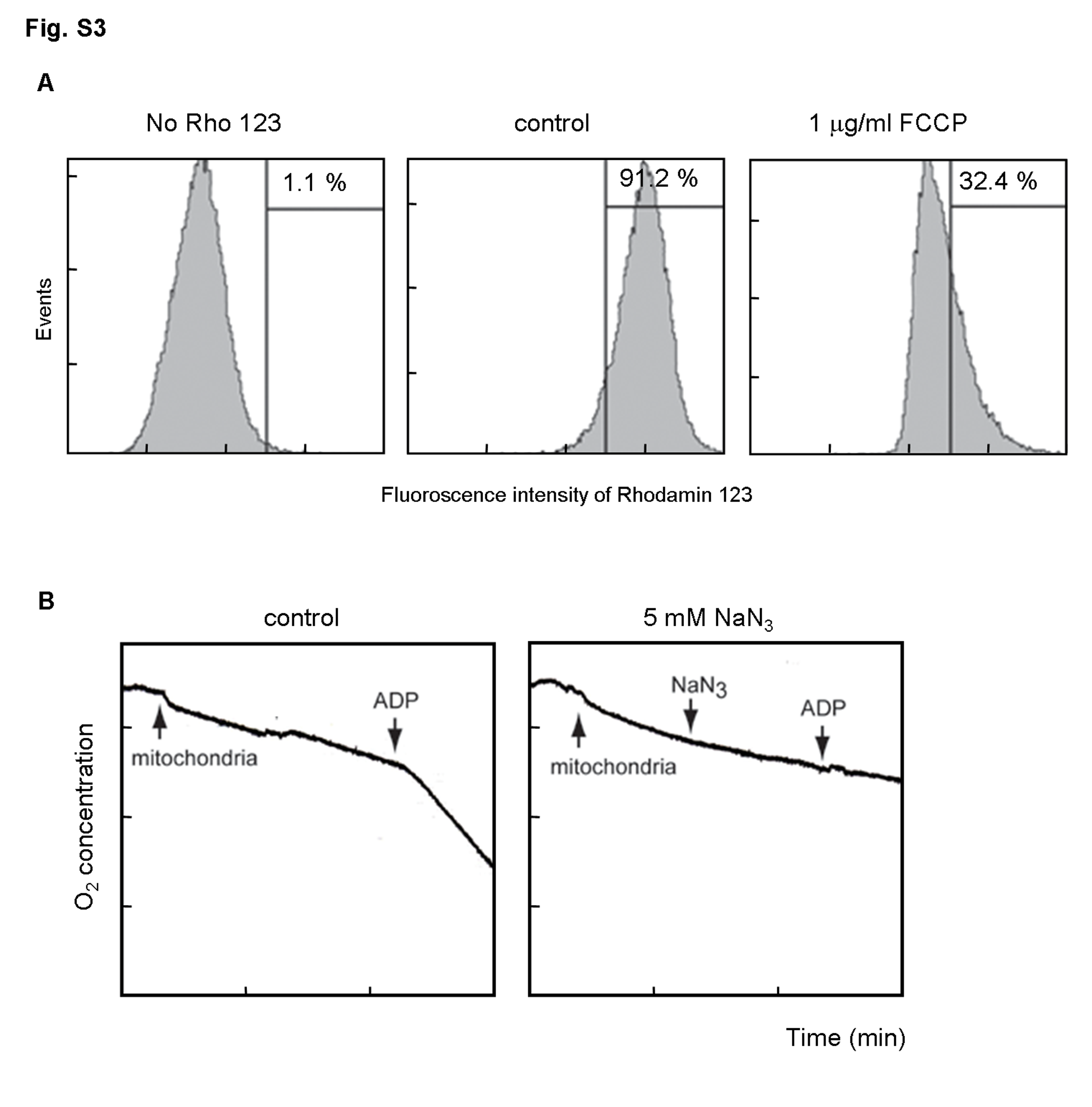

Supplement: Figure S3 — Analysis of mitochondria activity. (A) The membrane potential of mitochondria was determined by incorporation of Rhodamine 123 (Rho 123). The percentage of mitochondria above a fluorescence threshold is shown at the top right corner of each panel. (B) After incubation of mitochondria (mito) with 5 mM NaN3 for 5 min, ADP was added, and oxygen concentration in solution was measured realtime. (5.64 MB TIF) [file pone.0003070.s003.tif]

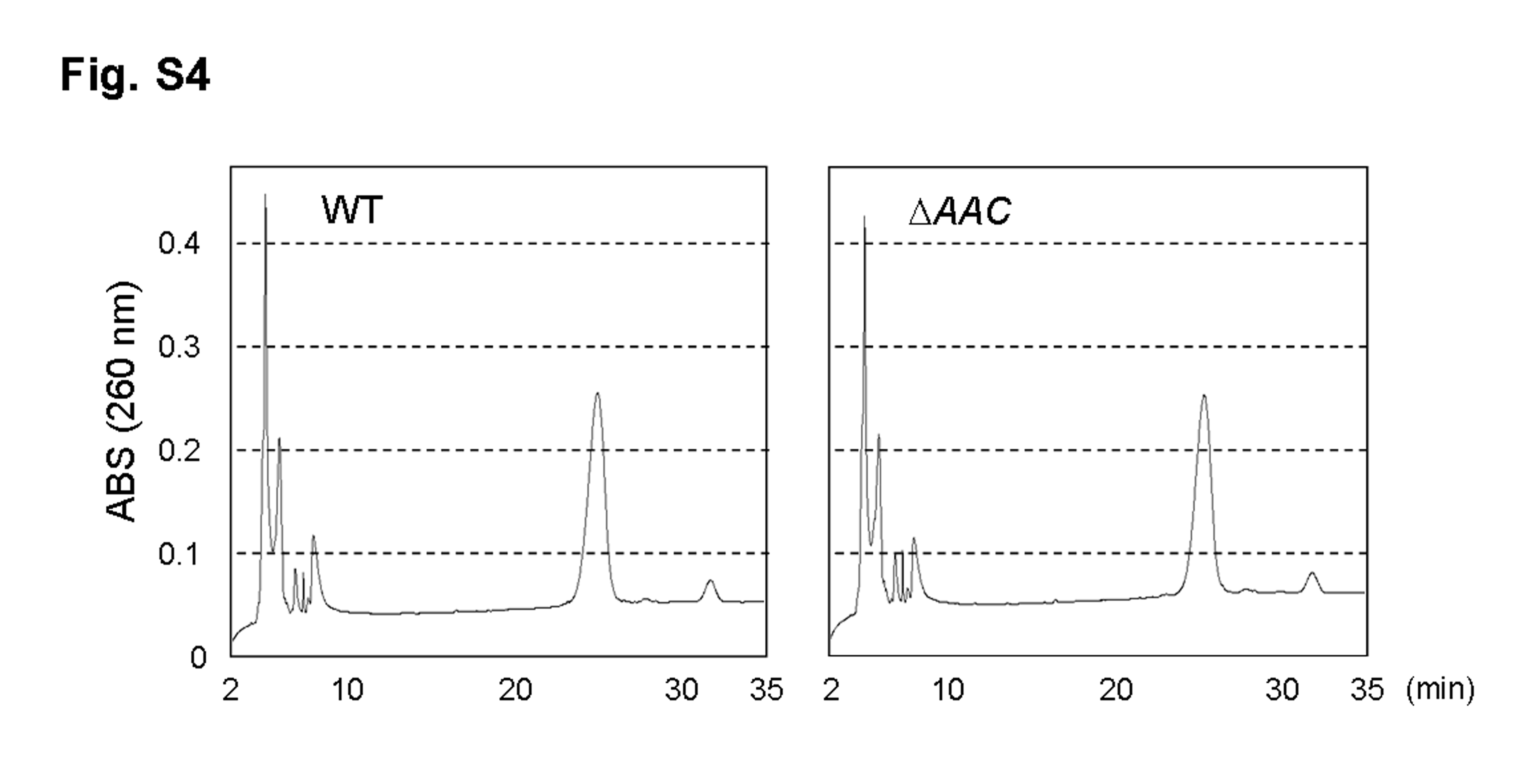

Supplement: Figure S4 — Absorbance of extracts from the WT or ΔAAC yeast strain. Extracts from the WT or ΔAAC yeast strain used in Fig. 4C were analyzed by C18 reverse phase HPLC and the absorbance of the eluted fractions was measured at 260 nm. (1.47 MB TIF) [file pone.0003070.s004.tif]

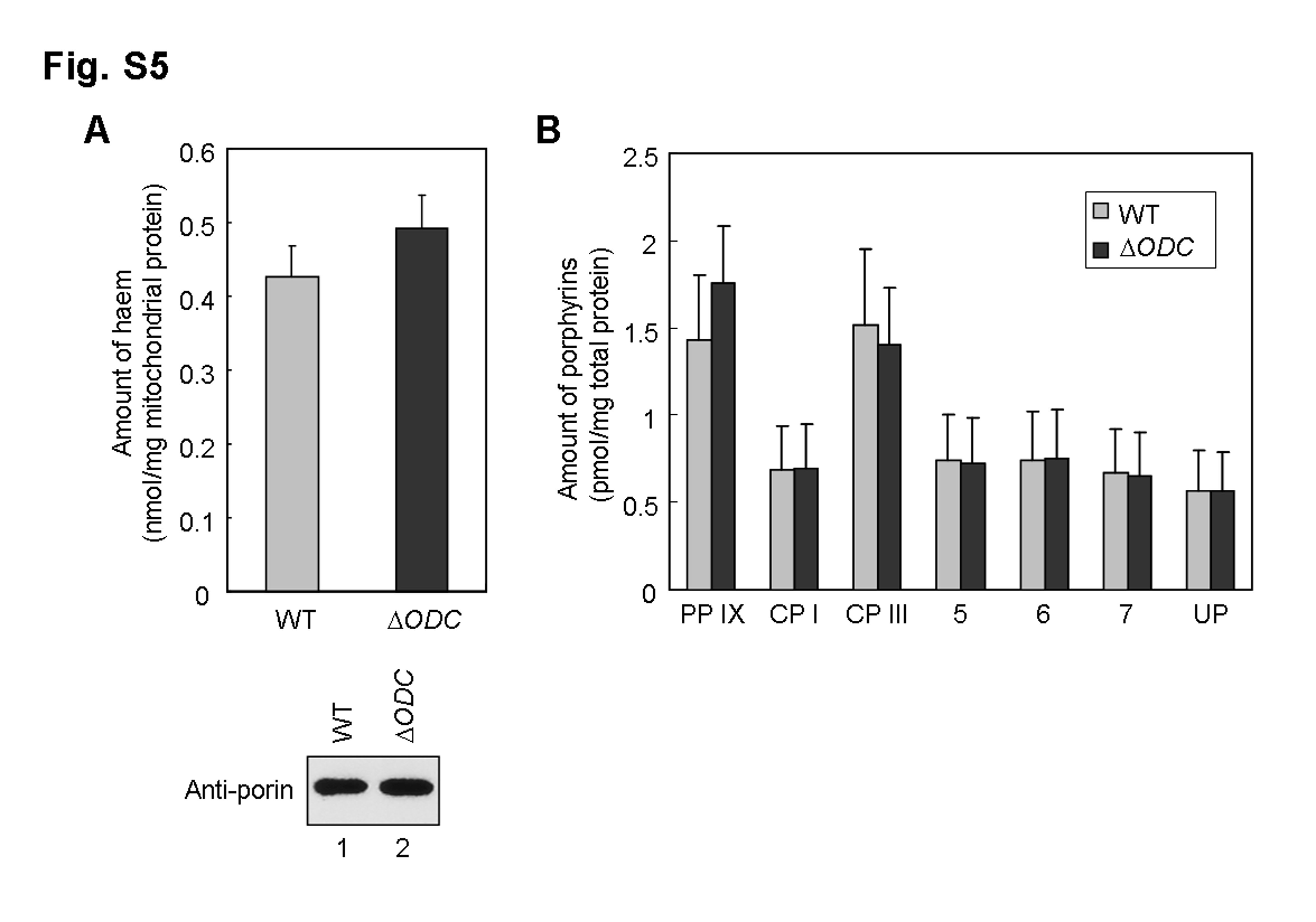

Supplement: Figure S5 — Analysis of haem biosynthesis in the OGC-deficient yeast strain. (A) The amount of mitochondrial haem in the wild-type (WT) or ΔODC yeast strain was measured with a fluorometric detector as described in Materials and Methods. Lower panel shows Western blot analysis of mitochondrial extracts using anti-porin antibody. (B) The concentrations of haem precursors (UP, uroporphyrin; 7, heptaporphyrin; 6, hexaporphyrin; 5, pentaporphyrin; CP I, coproporphyrin I; CP III, coproporphyrin III; PP IX, protoporphyrin IX) in the WT or ΔODC yeast strain. Data represent mean±s.e.m. from five independent experiments. (2.47 MB TIF) [file pone.0003070.s005.tif]

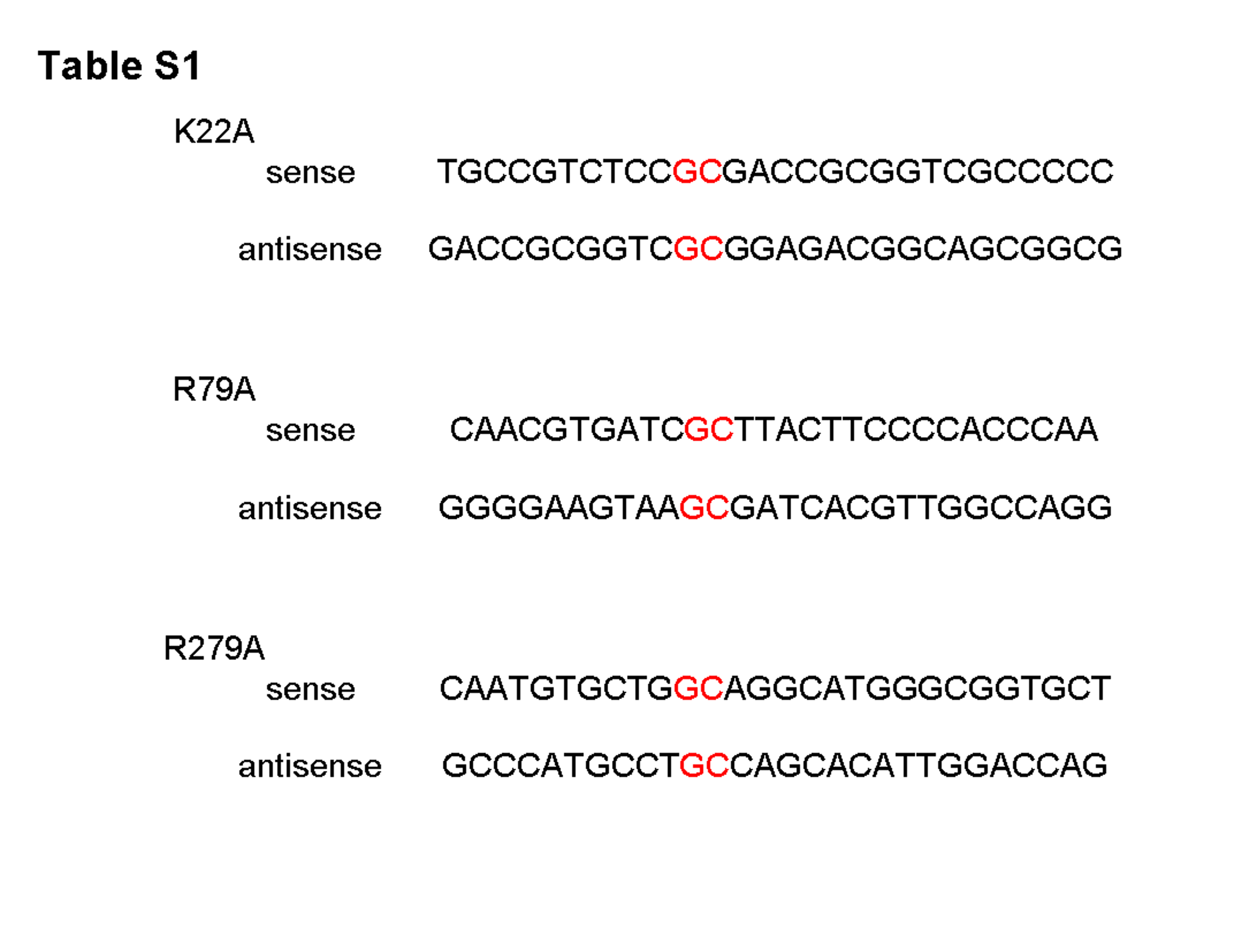

Supplement: Table S1 — (1.56 MB TIF) [file pone.0003070.s006.tif]
