# Supplementary material for: Adenine Nucleotide Translocator Transports Haem Precursors into Mitochondria
Source: PLoS One. 2008 Aug 27;3(8):e3070. doi: 10.1371/journal.pone.0003070 (PMC2516936; doi:10.1371/journal.pone.0003070)
Supplement: Text S1 — Supporting Method S1. (0.03 MB DOC) [file pone.0003070.s007.doc]

**Supporting information**

**Method S1**

**Analysis of mitochondria activities**

For analysis of mitochondrial membrane potential, rat liver mitochondria (1 mg/ml) were pretreated with or without 1 μg/ml FCCP in buffer (10 mM phosphate (pH 7.4), 150 mM sucrose, 20 mM KCl, 5 mM MgCl_2_, 5mM succinate) for 5 min at 30 ºC. Then, Rhodamin 123 was added a final concentration of 10 μM, and mitochondria were further incubated for 15min at at 30 ºC. Mitochondria were then washed with ice-cold buffer twice and analyzed using FACSAria (Becton–Dickinson, Tokyo, Japan). For analysis of oxygen consumption, rat liver mitochondria (1 mg/ml) were pretreated with or without 1 μg/ml NaN_3_ in buffer for 5 min at room temperature. Then, ADP was added to final concentration of 400 μM, and oxygen electrode with continuous stiring [28].
